# Cross-sectional analysis of the association between information and communication technology and mental health among Korean workers

**DOI:** 10.1371/journal.pone.0310248

**Published:** 2024-11-04

**Authors:** Yeona Shin, Seunghyun Lee, Wanhyung Lee

**Affiliations:** 1 Incheon Environmental Health Center, Incheon, Republic of Korea; 2 Department of Convergence Medicine, School of Medicine, Pusan National University, Yangsan, Republic of Korea; 3 Department of Preventive Medicine, College of Medicine, Chung-Ang University, Seoul, Republic of Korea; Sunway University, MALAYSIA

## Abstract

**Background:**

The adoption of information and communication technology (ICT) has been the fastest and most widespread in the South Korean workplace. While ICT has several advantages, it can also cause stress among workers. However, the relationship between the introduction of ICT in the workplace and mental health problems among Korean workers remains unclear.

**Methods:**

We conducted a cross-sectional analysis of data obtained from the sixth Korean Working Conditions Survey (KWCS). In total, 40,019 participants answered a question about whether ICT had been introduced in their workplace. Among these, we compared the rate of complaints of mental health disorders between those who answered “yes” (n = 3,250) and those who answered “no” (n = 36,769). We analyzed the data using the SAS statistical software and calculated the odds ratios (ORs) and 95% confidence intervals (95% CIs) for anxiety, insomnia, and depression using multiple logistic regression models. We also stratified the data on Korean workers to assess the impact of ICT on subgroups.

**Results:**

In our nationally representative cohort, on experiencing ICT incorporation at work, 384 participants (11.8%) reported anxiety, 138 (4.2%) reported insomnia, and 296 (9.1%) reported depression. In contrast, among participants who did not have access to ICT, 1,929 (5.2%) reported anxiety, 702 (1.9%) reported insomnia, and 4,404 (12.0%) reported depression. The ORs (95% CIs) for anxiety and insomnia complaint rates were 2.47 (2.19–2.79) and 2.55 (2.10–3.10), respectively, among workers who experienced new ICT adaptations in comparison with those who did not. However, no significant relationship was observed between ICT adoption and depression.

**Conclusions:**

The causes of mental health problems in the workplace should be identified and addressed. We found that the introduction of ICT in the workplace was significantly related to anxiety and insomnia symptoms among Korean workers, after controlling for the selected covariates. This information can be used to identify subgroups in the workplace that are vulnerable to ICT changes and tailor interventions to their social and demographic profiles.

## Introduction

The global landscape of information and communication technology (ICT) has undergone rapid and profound changes over the past few decades, reshaping workplaces worldwide. In this global context, South Korea has consistently been among the top 3 countries in the ICT Development Index, ranking second globally in 2016 [1]. As reported by Kim et al. (2022), digital transformation in South Korea has been more rapid and comprehensive than that in many of its global counterparts [2]. The development of ICT in Korea has been extensive, with foundational support beginning as early as the 1980s [3]. The Korean government established a framework for the IT industry and accelerated efforts to enhance digital literacy and internet access [4]. In particular, Korea’s implementation of the National Informatization Master Plan in the late 1990s facilitated the transition toward e-government services, addressing the challenges of rapid digitalization [3]. By the 2010s, ICT permeated Korean workplaces, revolutionizing operational practices with smart technologies and digital platforms [5]. By the 2020s, the focus shifted toward implementing smart technologies and fostering remote work solutions, greatly transforming workplace practices. Moreover, organizations increasingly adopted digital platforms that enhanced collaboration and productivity [6].

Although this digital transformation significantly improved efficiency and collaboration across various sectors, it also had adverse effects on workers [7]. Early studies in the 2000s focused on the fact that the introduction of ICT in the workplace increased job losses with an increase in productivity [8]. Recent research has shifted toward examining the multifaceted impacts of ICT on the physical and mental health of workers [7, 9].

Mental health issues among Korean workers encompass a wide spectrum of disorders, such as anxiety, sleep disturbances, and depression, and more severe conditions, such as bipolar disorder and post-traumatic stress disorder (PTSD) [10]. The prevalence of these issues has shown a concerning increase over time. According to the Korean Working Conditions Survey (KWCS), less than 10% of workers reported experiencing depression or anxiety in 2014, while in 2020, nearly half of workers reported experiencing anxiety [11, 12]. This increasing trend underscores the growing mental health crisis in the Korean workforce, potentially exacerbated by the pervasive use of ICT in the workplace.

Several ICT-related factors contribute to the deterioration of mental health among workers. The implementation of new technologies often increases work complexity, job uncertainty, and job insecurity [13]. Moreover, the culture of constant connectivity enabled by digital devices has created an “always on” work environment, which is associated with work overload and the invasion of personal life, resulting in heightened stress levels [14]. Furthermore, the pressure to continuously update technical skills to keep pace with rapidly evolving technologies increases the workload and stress experienced by employees [15]. This study assessed the prevalence of anxiety, sleep disorders, and depression among Korean workers and determined the impact of integrating ICT in the workplace on these mental health issues at the national level. Moreover, it analyzed whether the deployment of new ICT tools has differential effects on various employee demographics.

## Materials and methods

### Data collection and study population

**Fig 1:**
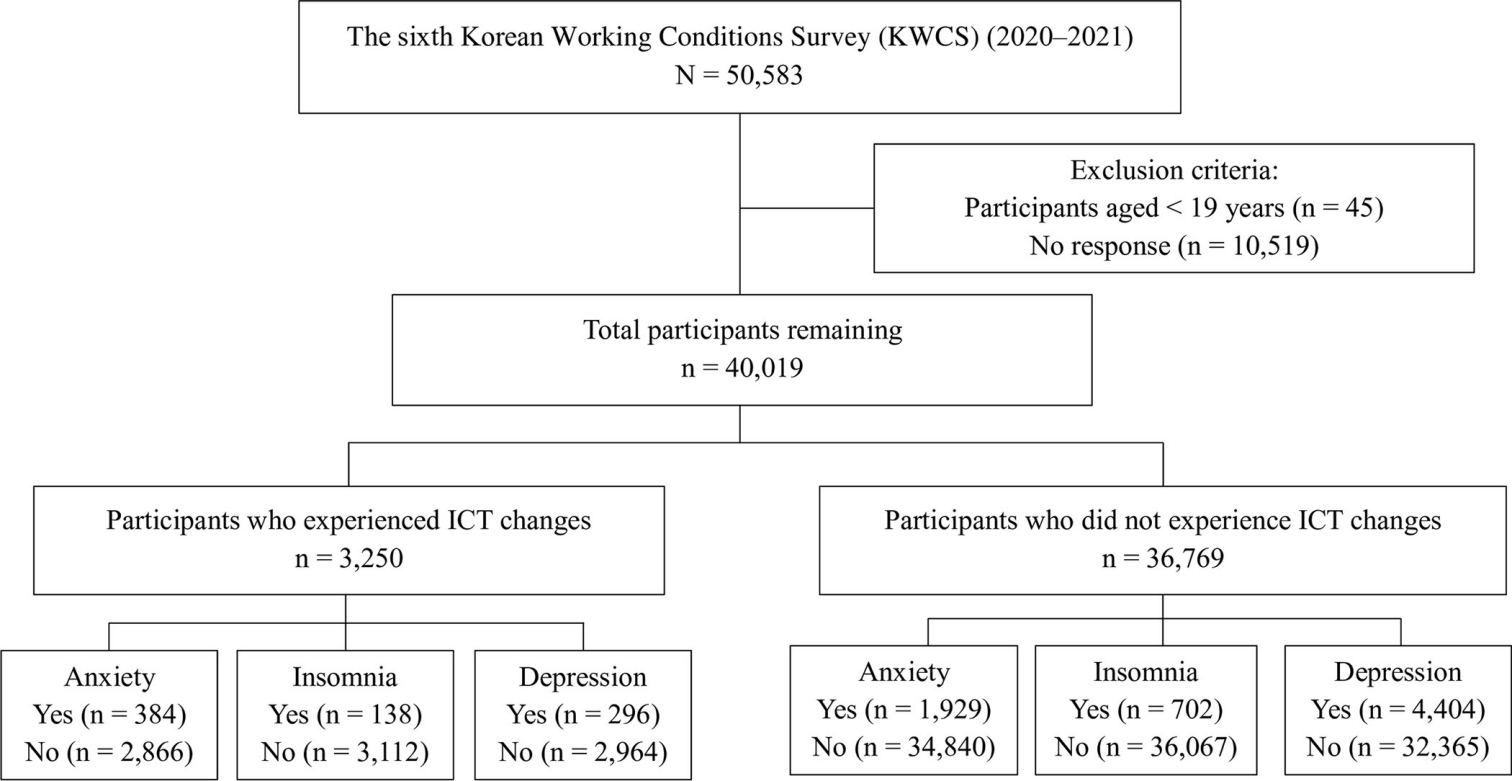
The schematic diagram of study participants.

Data for this study were collected from the KWCS, which serves as a valuable resource for researchers, policymakers, and organizations seeking to understand and improve working conditions in Korea [16]. The KWCS has benchmarked its questionnaires using the European Working Conditions Survey (EWCS) by Eurofound. First implemented in 2006, the KWCS is periodically conducted to monitor changes in the Korean labor market and working environments. The sixth KWCS, in which data were obtained from a nationally representative sample of workers across various sectors and occupations, was utilized in this study. This survey covers a wide range of topics, including physical and psychosocial work environments, work organization, work–life balance, and occupational health and safety. It provides robust, nationally representative data that facilitates the examination of trends and relationships between various aspects of work and worker well-being [17]. Trained interviewers conducted a 1:1 survey with 50,583 individuals. After excluding missing data from the target questionnaires, 40,019 participants were included in the final study sample. The process of study selection is presented as a flow chart in Fig 1.

### Main variables

A recent survey question in the sixth KWCS was used in this study: “During the last 3 years (or since you started your job), have new or significantly changed information and communication tools (including computers, mobile phones, software, apps, and networks) been introduced at your company or organization?” The possible responses were “yes” or “no.” The participants also answered questions regarding mental health disorders, including whether they had experienced anxiety, insomnia, or depression because of their jobs. Anxiety complaints were investigated through the following question: “Over the last 12 months, did you have any of the following health problems: Anxiety?” The possible responses were “yes” or “no” [18, 19]. Insomnia symptoms were defined using the Minimal Insomnia Symptom Scale (MISS) [20], which is psychometrically sound and can provide reliable results for adults [21]. The MISS is a simple insomnia screening tool that comprises the following three categories: “Difficulty falling asleep,” “Waking up repeatedly during sleep,” and “Waking up with a feeling of exhaustion and fatigue.” Each response is scored as follows: daily = 4, several times a week = 3, several times a month = 2, rarely = 1, and never = 0 [20]. The total raw score ranges from 0 to 12, with a total score of ≥9 indicating insomnia [20]. Depression was assessed using the World Health Organization Five Well-Being Index (WHO-5), which is a globally used tool for measuring subjective well-being [22, 23]. The WHO-5 includes the following statements to assess the participants’ mental well-being: “I have felt cheerful and in good spirits,” “I have felt calm and relaxed,” “I have felt active and vigorous,” “I wake up feeling fresh and rested,” and “My daily life has been filled with things that interest me.” The responses are rated as follows: all of the time = 5, most of the time = 4, more than half of the time = 3, less than half of the time = 2, some of the time = 1, and at no time = 0. The total raw score, ranging from 0 to 25, is multiplied by 4 to obtain the final score [22]. In this study, the baseline mean WHO-5 score was below the cut-off score for clinical depression (≤28) [22].

### Covariates

The following possible confounders were selected by reviewing previous reports: sex, age, education level, household income, job classification, weekly working hours, and shift work [15]. The age groups included younger age (≤35 years), middle age (36–55 years), and older age (>55 years), according to the Korean Statistical Information Service (KOSIS). Education level was divided into three categories: middle school or lower, high school, and college or higher. Household income was categorized according to the quartiles of total household income. Occupations were divided into six categories: executive white collar (legislators, senior officials, managers, and professionals), ordinary white collar (technicians and associated professionals), pink collar (clerks, salespersons, and customer service workers), green collar (agriculture, fishery, and forestry workers), skilled blue collar (craft persons, plant and machinery operators, and assemblers), and unskilled blue collar (elementary workers). Working hours per week were categorized as less than 41 hours, 41–52 hours, and more than 52 hours. Finally, whether the working schedule of the participants was shift work was assessed. The term “shift work” refers to non-daytime work, including irregular or rotating schedules and evening and night work [24].

### Statistical analysis

Data were analyzed using the SAS statistical software (version 9.4; SAS Institute Inc., Cary, NC, USA), and chi-square tests were performed to compare differences in baseline characteristics between the control and case groups. Moreover, odds ratios (ORs) and 95% confidence intervals (95% CIs) for anxiety, insomnia, and depression among workers based on their experiences of working with the newly introduced ICT were estimated using logistic regression models adjusted for sex, age, education level, household income, job classification, weekly working hours, and shift work. The fit of the model was estimated using backward stepwise logistic regression, and a stratified analysis was conducted to check for effect modifications.

### Ethical approval and consent to participate

This study was performed in accordance with the ethical standards of the Declaration of Helsinki (1964) and its subsequent amendments. The KHPS data were anonymized before their release to the authors. All participants provided written informed consent. This study was approved by the Institutional Review Board of the Gil Medical Center, Gachon University (IRB number: GFIRB2019-278).

## Results

Of the 40,019 participants, 3,250 (8.1%) experienced new ICT adaptations at their company or organization within 3 years; these included 30,962 men (77.4%) and 9,057 women (22.6%; Table 1). In the study sample, the highest proportion of workers who experienced new ICT adaptations belonged to the highest household income group (16.4%), followed by executive white-collar occupation (15.6%), ordinary white-collar occupation (12.8%), and higher education level (12.2%) groups. As shown in Table 1, the prevalence of anxiety and insomnia complaints was higher among workers who experienced new ICT adaptations than among those who did not. On the other hand, the prevalence of depression was similar between workers who experienced ICT changes and those who did not.

**Table 1:** The baseline characteristics of workers who experienced ICT changes at their workplace within 3 years.

Characteristics		Total (n = 40,019)	Workers who experienced ICT changes at their workplace, n(%)		p-value
			Yes (n = 3,250)	No (n = 36,769)	
Sex					
	Male	30,962	2,688 (8.7%)	28,274 (91.3%)	<0.0001
	Female	9,057	562 (6.2%)	8,495 (93.8%)	
Age group					
	Younger age (≤35 years)	8,888	866 (9.7%)	8,022 (90.3%)	<0.0001
	Middle age (36–55 years)	15,022	1,602 (10.7%)	13,420 (89.3%)	
	Older age (>55 years)	16,109	782 (4.9%)	15,327 (95.1%)	
Education level					
	Middle school or below	2,785	43 (1.5%)	2,742 (98.5%)	<0.0001
	High school	15,958	614 (3.9%)	15,344 (96.1%)	
	College or higher	21,276	2,593 (12.2%)	18,683 (87.8%)	
Household income					
	First quartile	10,429	276 (2.7%)	10,153 (97.3%)	<0.0001
	Second quartile	13,508	850 (6.3%)	12,658 (93.7%)	
	Third quartile	9,040	968 (10.7%)	8,072 (89.3%)	
	Fourth quartile	7,042	1,156 (16.4%)	5,886 (83.6%)	
Job classification					
	Executive white collar	7,870	1,231 (15.6%)	6,639 (84.4%)	<0.0001
	Ordinary white collar	7,342	937 (12.8%)	64050 (87.2%)	
	Pink collar	12,926	487 (3.8%)	12,439 (96.2%)	
	Green collar	1,443	32 (2.2%)	1,411 (97.8%)	
	Skilled blue collar	7,207	477 (6.6%)	6,730 (93.4%)	
	Unskilled blue collar	3,231	86 (2.7%)	3,145 (97.3%)	
Shift work					
	Yes	3,008	306 (10.2%)	2,702 (89.8%)	<0.0001
	No	37,011	2,944 (8.0%)	34,067 (92.0%)	
Weekly working hours					
	≤40	23,928	2,248 (9.4%)	21,680 (90.6%)	<0.0001
	41–52	9,613	765 (8.0%)	8,848 (92.0%)	
	>52	6,478	237 (3.7%)	6,241 (96.3%)	
Anxiety					
	Yes	2,313	384 (16.6%)	1,929 (83.4%)	<0.0001
	No	37,706	2,866 (7.6%)	34,840 (92.4%)	
Insomnia					
	Yes	840	138 (16.4%)	702 (83.6%)	<0.0001
	No	39,179	3,112 (7.9%)	36,067 (92.1%)	
Depression					
	Yes	4,700	296 (6.3%)	4,404 (93.7%)	<0.0001
	No	35,319	2,954 (8.4%)	32,365 (91.6%)	

Table 2 presents the association between mental health disorder complaint rates and the introduction of ICT. The data were adjusted for sex, age, education level, income, occupation, weekly working hours, and shift work. Individuals who experienced the implementation of ICT had more anxiety (OR [95% CI]: 2.58 [2.29–2.92]) and insomnia (OR [95% CI]: 2.55 [2.09–3.10]) than those who did not (p < 0.0001). However, no correlation was noted between ICT changes and depression complaints (OR [95% CI]: 0.97 [0.85–1.10]).

**Table 2:** Results of multiple logistic regression for mental health problems according to ICT changes in the workplace.

ICT changes	Mental health problems, odd ratio (95% confidence interval)		
	Anxiety	Insomnia	Depression
Yes	2.58 (2.29–2.92)	2.55 (2.09–3.10)	0.97 (0.85–1.10)
No	Reference	Reference	Reference

Adjusted for sex, age, education level, household income, occupation, weekly working hours, and shift work.

S1 Table presents the mental health issue complaint rates depending on individuals’ perception of ICT changes. Individuals who held negative views toward ICT changes had a higher incidence of anxiety, insomnia, and depression than those who viewed such changes positively or neutrally.

S2 Table presents specific data regarding anxiety, insomnia, and depression complaints among the different groups of workers who experienced ICT changes. With regard to sex, both male and female workers who experienced ICT changes exhibited increased anxiety and insomnia complaint rates. No correlation was noted between ICT changes and depression complaints among male workers; however, female workers exhibited slightly lower levels of depression. With regard to age, workers in all age groups who experienced ICT changes were more likely to report anxiety and insomnia than those who did not. However, no such relationship was noted between depression complaints in all age groups and ICT changes. With regard to education level, workers with a high school diploma, college degree, or higher degree who experienced ICT changes at work had a significantly increased prevalence of anxiety and insomnia than those with lower levels of education, as shown in S2 Table. However, in the middle school or below and college or higher education level groups, the depression complaint rates were similar between workers who experienced ICT changes and those who did not. In terms of household income, all quartiles exhibited increased anxiety and insomnia complaint rates when they experienced ICT changes in the workplace. Moreover, in all quartiles, no correlation was noted between ICT changes and depression complaints. With regard to job classification, executive white-collar, ordinary white-collar, and skilled blue-collar workers exhibited higher anxiety and insomnia complaint rates resulting from ICT implementation. However, no significant association was noted between depression complaints and ICT adaptation among executive white-collar and ordinary white-collar workers. Pink-collar workers only reported anxiety complaints because of ICT changes in the workplace. Moreover, their depression rate was lower when they experienced ICT changes at work. As shown in S2 Table, no correlation was noted between mental health disorders and ICT changes among green-collar and unskilled blue-collar workers. Moreover, analysis of the mental health of workers based on weekly working hours and shift work revealed that anxiety and insomnia symptoms were significantly associated with the implementation of ICT in groups of workers with less than 40 hours, 41–52 hours, and more than 52 hours of weekly work and among those with shift and non-shift work. On the other hand, no correlation was noted between ICT changes and depression complaint rates regardless of weekly working hours and shift or not-shift work.

## Discussion

Our nationwide study is the first to assess whether the recent implementation of ICT in the workplace is correlated with mental health disorders, such as anxiety, insomnia, and depression, among Korean workers. We found that workers who experienced ICT changes in their workplace exhibited higher anxiety and insomnia complaint rates than those who did not. This correlation was not attenuated even after adjusting for sociodemographic characteristics and various working conditions, which were regarded to be possible confounders. These results may have several possible explanations.

### Work environment and work stress

According to previous studies, workers’ mental health is closely related to work stress. Too much information, work complexity, and the pressure to learn up-to-date skills quickly are considered the main sources of work stress [25]. Information overload, which workers cannot address efficiently, also increases their workload. This increased workload must be completed in the same amount of time, adding to the stress of having to work more quickly [26]. Moreover, if the newly implemented technology is complex, it may increase the difficulty of tasks, thereby worsening workers’ stress levels and mental health [26]. Learning new skills with the introduction of ICT can add to the workload, and the pressure and stress of trying not to be left behind can trigger anxiety and insomnia symptoms among workers [27, 28]. For instance, job classifications that demand high connectivity through ICT may increase anxiety and insomnia symptoms because of excessive workloads and performance pressures [29]. However, these stressors may not necessarily correlate with symptoms of depression if the work environment provides adequate support and resources [30]. Under supportive conditions, employees may cope with ICT-related demands without experiencing depression, leading to a situation where anxiety and insomnia are heightened but depressive outcomes remain unaffected [30].

### Communication overload and psychological stress

The phenomenon of communication overload associated with ICT adoption significantly increases anxiety and insomnia [25]. Constant notifications and the expectation of immediate responses can create an environment where workers feel perpetually on edge [31]. Such relentless engagement can cause sleep disruptions as workers struggle to disconnect from their tasks and responsibilities, fostering insomnia [25]. Moreover, workers may feel that some of these social interactions are irrelevant and time-consuming, which may result in anxiety, exhaustion, and burnout [32]. Privacy is essential for workers to rest and unwind. When their private time is interrupted, workers are deprived of the opportunity to recover from stress; a prolonged invasion of privacy can also induce anxiety and sleep disorder issues [33]. However, the psychological burden of this communication overload may not always culminate in depression if individuals can manage their stress effectively or find fulfillment in their work through technology [30].

### Work unpredictability and mental health

Workers may find the newly introduced ICT unpredictable and ambiguous because of its rapid evolution, complex nature, and varied implementation across workplaces. This unpredictability often stems from constant updates, technical issues, and the challenge of integrating new technologies into existing work processes, which can leave workers feeling uncertain and stressed about their ability to adapt and perform effectively [34]. Workers are also frustrated when they are unsure of what to do with ICT changes. Moreover, the fear of changes in the working environment can cause anxiety and sleep disorders [35, 36]. For the stressors mentioned above, researchers have explained that when stress increases beyond one’s control, one often forgets “knowledge obtained on stress or how to effectively manage it,” which results in feelings of anger and frustration. In extreme cases, this can also cause depression [35].

### Individual resilience and coping mechanisms

Individuals can perceive an ICT change as stressful or threatening depending on their personal traits. Individual differences, such as resilience and coping strategies, may influence how each individual experiences ICT-related pressures [29]. In contrast to individuals who view a change positively or neutrally, those who view it negatively may experience negative impacts on their mental well-being when required to adopt the change [37]. Employees who exhibit higher resilience are more likely to adopt positive attitudes toward technology, allowing them to manage ICT-related stressors effectively [30, 38]. This adaptive response can help mitigate the risk of experiencing depression while still experiencing anxiety or insomnia due to technological demands [38]. Consequently, resilient workers may navigate the complexities of ICT without succumbing to depression, even when they experience anxiety and compromised sleep quality [30].

### Demographic factors

Previous research has identified that the same stressor of change in the working environment can affect workers differently depending on their sex, age, occupation, and other factors. Therefore, this study also assessed whether the mental health of different subgroups of workers is significantly correlated with the introduction of ICT in the workplace (S2 Table). Male and female workers who experienced ICT changes reported higher anxiety and insomnia than those who did not. The effects of sex on ICT-related depression are complex. Marchiori et al. (2019) explained that men are primarily affected by ICT-induced work overload and life invasion, whereas women are influenced by techno-complexity and uncertainty [13]. Cooper (2006) suggested that sex-related stereotypes regarding technological competence disproportionately affect women, potentially increasing their stress levels [39]. Moreover, Martinengo et al. (2010) highlighted the role of work–life balance, suggesting that ICT changes blurring work–life boundaries have a more pronounced effect on women because they often have greater domestic responsibilities [40]. Ragu-Nathan et al. (2008) proposed that sex-related differences in coping strategies can influence susceptibility to technostress and related symptoms of depression; however, more research is needed to elucidate these mechanisms [39].

Stratified analyses by age revealed that workers in all age groups experienced the detrimental effects of ICT changes in their workplace. Although older age groups are regarded as having better stress management skills and maturity [25, 41], workers in these age groups found the new technology complicated and stressful [13, 25]. In the middle and younger age groups, workers aged between 35 and 45 years exhibited the highest mental health disorders due to ICT. In these workers, technostress may worsen when combined with other stressors, such as family care and career progression [25]. Although younger age groups have better skills in dealing with new technology [13, 39, 42], their mental health may be more vulnerable to changes in work tasks and occupational environments [39, 42, 43].

Analyses by education level revealed that workers with a high school diploma reported all mental health issues, while those with a college degree or higher reported higher anxiety and insomnia symptoms when they had to adopt ICT in their workplace. This may be because individuals with higher levels of education are frustrated by handling ICT breakdowns, and their negative perceptions of technostress creators cause mental health problems [44].

Analyses based on household income category revealed increased anxiety and insomnia in all quartiles of household income. Workers with a low income generally have fewer opportunities to experience ICT, and the rapid pace of ICT changes counterbalances their stress [45]. On the other hand, workers with a higher income tend to have better education and skills; however, if they encounter any minor techno-induced issue, it may negatively impact their income, technological efficiency, and self-efficacy and cause mental health issues [46].

Stratified analyses by occupation revealed that workers in some job categories who experienced ICT changes reported more mental health complaints than those who did not. White-collar workers and ordinary white-collar workers with a low level of job control and a high level of psychological demand tend to be more susceptible to ICT changes. Their stress stems from various issues, such as information overload, the disruption of work–life balance due to constant connectivity, and difficulties in separating professional and personal time [47]. In skilled blue-collar workers, mental health was found to be closely associated with ICT changes. These workers are highly trained in their current job conditions. If any innovation occurs, their enterprise-specific abilities become obsolete, resulting in stress and anxiety [48]. Moreover, the disconnect between traditional work practices and modern technology can exacerbate feelings of alienation because workers find it challenging to integrate new systems into established workflows [48]. Among pink-collar workers, only anxiety was associated with ICT changes in the workplace. While ICT has reduced opportunities for face-to-face interactions among service workers, which are often considered to reduce job stress, the pressure to learn new skills may increase anxiety rates [15].

Weekly working hours and shift work, which are widely recognized stressors affecting mental health diseases, were also considered in this study. In shift workers and employees working more than 52 hours a week, which is strongly associated with sleep disturbance, no significant association was noted between ICT changes and mental health. This suggests that the impact of working patterns is more important than changes in technology [49].

Our findings should be interpreted with caution because of some limitations. First, our analysis was based on cross-sectional data, which does not allow us to fully clarify the causal relationship between ICT and mental health problems. Second, the survey relied on self-reported data regarding employees’ perceptions of work-related mental health disorders, which may have been biased by individual personality traits. Third, a question regarding ICT changes in the workplace within 3 years was used in our study, which may have a risk of recall bias. Previous research has revealed that individuals who have experienced health problems are likely to overestimate their past health in personal retrospective surveys [50]. Prospective longitudinal studies that overcome the weaknesses of retrospective reporting are warranted to extend the findings of this study and investigate the psychosocial and biological causes of mental health issues.

Despite these limitations, our findings are novel because our study used a nationwide survey of workers, which represents the behavior of Korean wage workers in response to ICT changes. To the best of our knowledge, this is the first study to assess the impact of ICT implementation on different groups of workers from different perspectives. This comprehensive study allows us to assess the impact of ICT changes in the workplace on different groups of Korean workers. The findings significantly inform theoretical frameworks regarding the psychological impacts of technological changes in the workplace, particularly highlighting the phenomenon of technostress that arises from ICT use. These findings necessitate the integration of technostress concepts into existing workplace stress models to explain how technology can induce anxiety, sleep disorders, and cognitive strain among employees. Theories on employee well-being are warranted to balance the duality of efficiency and productivity gains resulting from technology against its potential to create psychological burdens [51]. This alignment can encourage organizations to develop holistic strategies for addressing these disparities while promoting healthy technology usage [52].

## Conclusions

In this study, a strong association was noted between the implementation of ICT in the workplace and mental health issues among Korean workers. While ICT offers numerous benefits, our findings indicated that workplace ICT changes correlate with increased anxiety and insomnia symptoms. Notably, workers who perceived ICT alterations as detrimental were more likely to report mental health concerns than those who viewed such changes as favorable or neutral. In our analysis of various worker subgroups, ICT affected all demographic subgroups based on sex, age, and household income; however, the effects varied based on education level, occupation, weekly work hours, and shift work patterns. These variations suggest that ICT changes manifest as diverse sources of job-related stress across different worker categories. These findings can serve as a valuable foundation for identifying vulnerable subgroups within the workforce and developing targeted interventions. Furthermore, our findings underscore the need for future research to explore and evaluate intervention programs tailored to mitigate the mental health impacts of ICT changes on specific workforce subgroups, ultimately contributing to a more holistic approach for promoting employee well-being in an increasingly digitized work environment.

## Supporting information

S1 Table OR (95% CI) of mental health issue complaint rates according to individual perspective toward ICTs change. (DOCX)

S2 Table ORs (95% CIs) for the relationship between mental health issue complaint rates among workers and ICT changes in the workplace. (DOCX)
