# Supplementary material for: Cross-sectional analysis of the association between information and communication technology and mental health among Korean workers
Source: PLoS One. 2024 Nov 4;19(11):e0310248. doi: 10.1371/journal.pone.0310248 (PMC11534220; doi:10.1371/journal.pone.0310248)
Supplement: S1 Table — (DOCX) [file pone.0310248.s001.docx]

**Table S1.** ORs (95% CIs) for mental health issue complaint rates according to individual perspectives toward ICT changes.

| **Individual perspectives toward ICT changes** | **Mental health issue complaint rates resulting from work stress** | | |
| --- | --- | --- | --- |
|  | **OR** | **95% CI** | **p-value** |
| **Negatively affected workers** |  |  |  |
| Anxiety | 2.19 | 1.43–3.34 | 0.0003 |
| Insomnia | 4.56 | 2.76–7.56 | <0.0001 |
| Depression | 2.85 | 1.85–4.39 | <0.0001 |
| **Positively affected or unaffected workers** |  |  |  |
| Anxiety | 0.46 | 0.30–0.70 | 0.0003 |
| Insomnia | 0.22 | 0.13–0.36 | <0.0001 |
| Depression | 0.35 | 0.23–0.54 | <0.0001 |
| Adjusted for sex, age, education level, household income, occupation, weekly working hours, and shift work. | | | |
