# Supplementary material for: Cross-sectional analysis of the association between information and communication technology and mental health among Korean workers
Source: PLoS One. 2024 Nov 4;19(11):e0310248. doi: 10.1371/journal.pone.0310248 (PMC11534220; doi:10.1371/journal.pone.0310248)
Supplement: S2 Table — (DOCX) [file pone.0310248.s002.docx]

**Table** **S2**. ORs (95% CIs) for the relationship between mental health issue complaint rates among workers and ICT changes in the workplace.

| **Mental health issues among**  **different worker groups** | | | **Workers who experienced ICT changes in their workplace** | | |
| --- | --- | --- | --- | --- | --- |
|  |  |  | **OR** | **95% CI** | **p-value** |
| Sex | |  |  |  |  |
|  | Male | |  |  |  |
|  |  | Anxiety | 2.69 | 2.35–3.08 | <0.0001 |
|  |  | Insomnia | 2.37 | 1.88–2.98 | <0.0001 |
|  |  | Depression | 1.09 | 0.95–1.25 | 0.2322 |
|  | Female | |  |  |  |
|  |  | Anxiety | 2.21 | 1.66–2.93 | <0.0001 |
|  |  | Insomnia | 3.24 | 2.20–4.77 | <0.0001 |
|  |  | Depression | 0.56 | 0.40–0.80 | 0.0011 |
| Age group | | |  |  |  |
|  | Younger age (≤35 years) | |  |  |  |
|  |  | Anxiety | 2.77 | 2.16–3.54 | <0.0001 |
|  |  | Insomnia | 2.31 | 1.55–3.43 | <0.0001 |
|  |  | Depression | 0.92 | 0.71–1.20 | 0.5324 |
|  | Middle age (36–55 years) | |  |  |  |
|  |  | Anxiety | 2.55 | 2.14–3.03 | <0.0001 |
|  |  | Insomnia | 2.31 | 1.73–3.09 | <0.0001 |
|  |  | Depression | 1.05 | 0.88–1.26 | 0.5689 |
|  | Older age (>55 years) | |  |  |  |
|  |  | Anxiety | 2.22 | 1.73–2.85 | <0.0001 |
|  |  | Insomnia | 2.97 | 2.07–4.27 | <0.0001 |
|  |  | Depression | 0.80 | 0.62–1.04 | 0.0894 |
| Education level | | |  |  |  |
|  | Middle school or below | |  |  |  |
|  |  | Anxiety | 1.39 | 0.42–4.60 | 0.5936 |
|  |  | Insomnia | 2.43 | 0.72–8.17 | 0.1518 |
|  |  | Depression | 0.64 | 0.25–1.64 | 0.3481 |
|  | High school | |  |  |  |
|  |  | Anxiety | 2.05 | 1.55–2.71 | <0.0001 |
|  |  | Insomnia | 2.92 | 1.97–4.33 | <0.0001 |
|  |  | Depression | 0.74 | 0.57–0.98 | 0.0343 |
|  | College or higher | |  |  |  |
|  |  | Anxiety | 2.73 | 2.38–3.14 | <0.0001 |
|  |  | Insomnia | 2.36 | 1.87–2.98 | <0.0001 |
|  |  | Depression | 1.04 | 0.90–1.21 | 0.5718 |
| Household income | | |  |  |  |
|  | First quartile | |  |  |  |
|  |  | Anxiety | 2.73 | 2.38–3.14 | <0.0001 |
|  |  | Insomnia | 2.36 | 1.87–2.98 | <0.0001 |
|  |  | Depression | 1.04 | 0.90–1.21 | 0.5718 |
|  | Second quartile | |  |  |  |
|  |  | Anxiety | 3.95 | 2.82–5.52 | <0.0001 |
|  |  | Insomnia | 2.98 | 2.11–4.22 | <0.0001 |
|  |  | Depression | 1.01 | 0.80–1.28 | 0.9273 |
|  | Third quartile | |  |  |  |
|  |  | Anxiety | 2.30 | 1.83–2.90 | <0.0001 |
|  |  | Insomnia | 2.84 | 1.94–4.18 | <0.0001 |
|  |  | Depression | 0.84 | 0.65–1.07 | 0.1601 |
|  | Fourth quartile | |  |  |  |
|  |  | Anxiety | 2.07 | 1.64–2.61 | <0.0001 |
|  |  | Insomnia | 1.70 | 1.16–2.50 | 0.0071 |
|  |  | Depression | 0.93 | 0.74–1.17 | 0.5411 |
| Job classification | | |  |  |  |
|  | Executive white collar | |  |  |  |
|  |  | Anxiety | 2.25 | 1.82–2.78 | <0.0001 |
|  |  | Insomnia | 2.35 | 1.70–3.25 | <0.0001 |
|  |  | Depression | 1.14 | 0.92–1.42 | 0.2261 |
|  | Ordinary white collar | |  |  |  |
|  |  | Anxiety | 3.41 | 2.71–4.28 | <0.0001 |
|  |  | Insomnia | 2.70 | 1.84–3.97 | <0.0001 |
|  |  | Depression | 1.06 | 0.84–1.34 | 0.6088 |
|  | Pink collar | |  |  |  |
|  |  | Anxiety | 2.09 | 1.54–2.83 | <0.0001 |
|  |  | Insomnia | 1.66 | 0.91–3.02 | 0.1003 |
|  |  | Depression | 0.64 | 0.44–0.92 | 0.0168 |
|  | Green collar | |  |  |  |
|  |  | Anxiety | 2.27 | 0.66–7.77 | 0.1913 |
|  |  | Insomnia | 2.57 | 0.58–11.38 | 0.2134 |
|  |  | Depression | 0.37 | 0.09–1.64 | 0.1971 |
|  | Skilled blue collar | |  |  |  |
|  |  | Anxiety | 2.43 | 1.76–3.36 | <0.0001 |
|  |  | Insomnia | 3.37 | 2.13–5.33 | <0.0001 |
|  |  | Depression | 0.68 | 0.49–0.94 | 0.0213 |
|  | Unskilled blue collar | |  |  |  |
|  |  | Anxiety | 1.99 | 0.94–4.23 | 0.0741 |
|  |  | Insomnia | 0.47 | 0.06–3.46 | 0.4599 |
|  |  | Depression | 0.91 | 0.49–1.70 | 0.7777 |
| Weekly working hours | | |  |  |  |
|  | ≤40 hours | |  |  |  |
|  |  | Anxiety | 2.42 | 2.06–2.85 | <0.0001 |
|  |  | Insomnia | 2.34 | 1.83–3.00 | <0.0001 |
|  |  | Depression | 0.89 | 0.76–1.05 | 0.1728 |
|  | 41–52 hours | |  |  |  |
|  |  | Anxiety | 3.22 | 2.59–4.00 | <0.0001 |
|  |  | Insomnia | 2.96 | 2.01–4.37 | <0.0001 |
|  |  | Depression | 1.24 | 0.98–1.56 | 0.0689 |
|  | >52 hours | |  |  |  |
|  |  | Anxiety | 2.05 | 1.38–3.03 | 0.0003 |
|  |  | Insomnia | 2.98 | 1.63–5.47 | 0.0004 |
|  |  | Depression | 0.82 | 0.55–1.22 | 0.3252 |
| Shift work | | |  |  |  |
|  | Shift work | |  |  |  |
|  |  | Anxiety | 1.62 | 1.04–2.50 | 0.0317 |
|  |  | Insomnia | 2.02 | 1.21–3.38 | 0.0074 |
|  |  | Depression | 0.71 | 0.46–1.09 | 0.1152 |
|  | Non-shift work | |  |  |  |
|  |  | Anxiety | 2.71 | 2.39–3.08 | <0.0001 |
|  |  | Insomnia | 2.63 | 2.13–3.26 | <0.0001 |
|  |  | Depression | 1.00 | 0.87–1.14 | 0.9440 |

Adjusted for sex, age, education, household income, occupation, weekly working hours, and shift work.
